# *catena*-Poly[[[aqua­(1,10-phenanthro­line)zinc(II)]-μ-3,3′-(*p*-phenyl­ene)di­acrylato] hemihydrate]

**DOI:** 10.1107/S1600536809025665

**Published:** 2009-07-18

**Authors:** Ya-Ping Li, Da-Jun Sun, Hu Zang, Guan-Fang Su, Yu-Lin Li

**Affiliations:** a Department of Ophthalmology, Second Hospital of Jilin University, Changchun 130041, People’s Republic of China; b Department of Vascular Surgery, China–Japan Union Hospital of Jilin University, Changchun 130033, People’s Republic of China; c Department of Orthopaedics, China–Japan Union Hospital of Jilin University, Changchun 130033, People’s Republic of China; d Teaching Laboratory of Pathology, Norman Bethune College of Medicine, Jilin University, Changchun 130041, People’s Republic of China

## Abstract

In the title compound, {[Zn(C_12_H_8_O_4_)(C_12_H_8_N_2_)(H_2_O)]·0.5H_2_O}_*n*_, each Zn^II^ atom is six-coordinated by two N atoms from one 1,10-phenanthroline (phen), three carboxyl­ate O atoms from two different *L* ligands [H_2_ *L* = 3,3′-(*p*-phenyl­ene)diacrylic acid] and one water mol­ecule in a distorted octa­hedral environment. The two *L* dianions are situated across inversion centres and bridge neighbouring Zn^II^ centres, yielding a chain propagating parallel to [100]. O—H⋯O hydrogen bonds between the coordinated water molecule, the solvent water molecule (half-occupied) and the carboxylate O atoms further stabilize the structure.

## Related literature

For general background and related structures see: Wang *et al.* (2008 ▶). For related literature, see: Batten & Robson (1998 ▶).
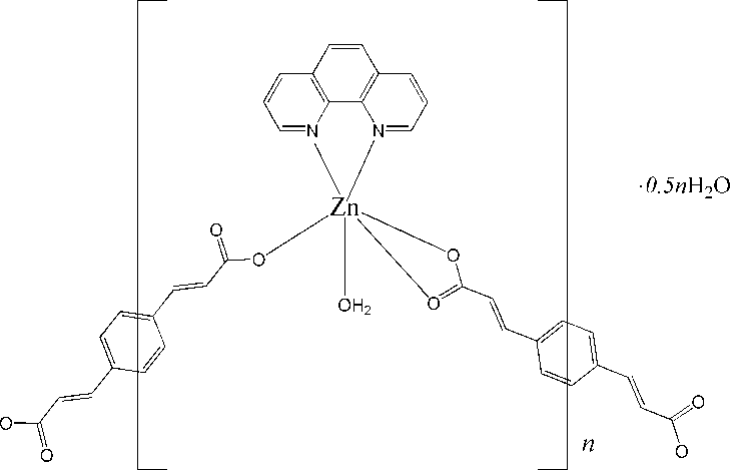


## Experimental

### 

#### Crystal data

[Zn(C_12_H_8_O_4_)(C_12_H_8_N_2_)(H_2_O)]·0.5H_2_O
*M* *_r_* = 488.78
Triclinic, 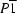

*a* = 8.959 (5) Å
*b* = 11.505 (5) Å
*c* = 11.691 (5) Å
α = 67.219 (5)°
β = 76.434 (5)°
γ = 89.555 (5)°
*V* = 1075.4 (9) Å^3^
*Z* = 2
Mo *K*α radiation
μ = 1.18 mm^−1^
*T* = 293 K
0.30 × 0.22 × 0.19 mm

#### Data collection

Bruker APEX CCD area-detector diffractometer
Absorption correction: multi-scan (*SADABS*; Sheldrick, 1996 ▶) *T*_min_ = 0.701, *T*_max_ = 0.792
6674 measured reflections
3887 independent reflections
3193 reflections with *I* > 2σ(*I*)
*R*_int_ = 0.019

#### Refinement

*R*[*F* ^2^ > 2σ(*F* ^2^)] = 0.036
*wR*(*F* ^2^) = 0.101
*S* = 0.98
3887 reflections
312 parameters
6 restraints
H atoms treated by a mixture of independent and constrained refinement
Δρ_max_ = 0.90 e Å^−3^
Δρ_min_ = −0.24 e Å^−3^

### 

Data collection: *SMART* (Bruker, 1998 ▶); cell refinement: *SAINT* (Bruker, 1998 ▶); data reduction: *SAINT*; program(s) used to solve structure: *SHELXS97* (Sheldrick, 2008 ▶); program(s) used to refine structure: *SHELXL97* (Sheldrick, 2008 ▶); molecular graphics: *SHELXTL* (Sheldrick, 2008 ▶); software used to prepare material for publication: *SHELXTL*.

## Supplementary Material

Crystal structure: contains datablocks global, I. DOI: 10.1107/S1600536809025665/at2836sup1.cif

Structure factors: contains datablocks I. DOI: 10.1107/S1600536809025665/at2836Isup2.hkl

Additional supplementary materials:  crystallographic information; 3D view; checkCIF report

## Figures and Tables

**Table 1:** Hydrogen-bond geometry (Å, °)

*D*—H⋯*A*	*D*—H	H⋯*A*	*D*⋯*A*	*D*—H⋯*A*
O1*W*—H*W*12⋯O2^i^	0.85 (3)	1.87 (3)	2.687 (3)	158 (4)
O1*W*—H*W*11⋯O3^i^	0.86 (2)	1.88 (3)	2.685 (3)	155 (3)
O2*W*—H*W*21⋯O2	0.816 (10)	2.044 (14)	2.856 (5)	173 (5)

Symmetry code: (i) 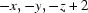.

**Fig. 1.**
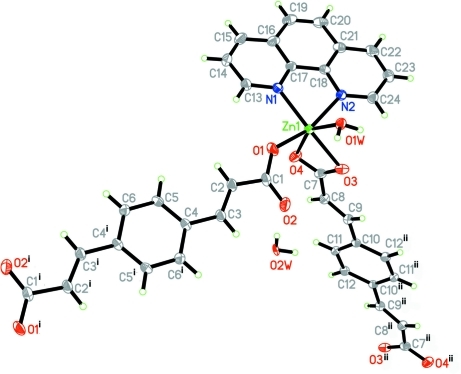
The asymmetric unit in the polymeric structure of the title compound, showing the atomic numbering scheme. Displacement ellipsoids are drawn at the 20% probability level [symmetry operations: (i) 1-x, -y, 3-z; (ii) -x-1, 1-y, 2-z].

**Fig. 2.**
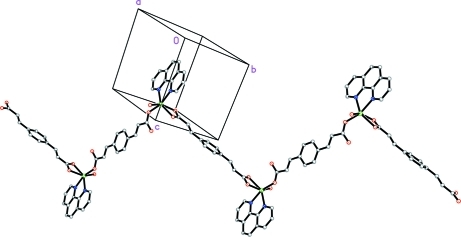
View of the one-dimensional chain of the title compound.

## supplementary crystallographic information

### Comment

Recently, chain structures have received much attention in coordination chemistry and medical chemistry (Batten & Robson, 1998). An appropriate bidentate organic acid bridge could be useful in the formation of chains in the presence of secondary ligands, such as 2,2'-bipyridine (bipy) and 1,10-phenanthroline (phen) (Wang *et al.*, 2008). The N atoms from the secondary ligand may occupy two coordination positions of the metal ions. The rest of the coordination positions are available for other carboxylate ligands to allow the formation of a chain. In this regard, 3,3'-(*p*-phenylene)diacrylic acid (H_2_L) is a good ligand in coordination chemistry because of its strong coordination ability and versatile coordination modes, so much attention has been paid to it in recent decades. In the present study, we selected H_2_L as a linker and phen as a secondary chelating ligand, forming a unique zigzag chain coordination polymer [Zn(phen)(*L*)(H_2_O)]^.^0.5H_2_O.

As shown in Fig. 1, each Zn^II^ atom is six-coordinated by two N atoms from one phen, three carboxylate O atoms from two different *L* ligands, and one water molecule in a distorted octahedral sphere. The two *L* dianions are situated across inversion centres. The bpea dianions bridge two neighboring Zn^II^ centres to form a one-dimensional chain (Fig. 2). The O—H···O_carboxylate_ H-bonding interactions further stabilize the structure of the title compound (Table 1).

### Experimental

A mixture of 3,3'-(*p*-phenylene)diacrylic acid (0.5 mmol), 1,10-phenanthroline (0.5 mmol), NaOH (1 mmol) and ZnCl_2_.2H_2_O (0.5 mmol) was suspended in deionized water (12 ml) and sealed in a 20 ml Teflon-lined autoclave. After heating at 438 K for one week, the autoclave was cooled slowly to room temperature. Crystals were collected, washed with deionized water and dried.

### Refinement

All H atoms were positioned geometrically (C—H = 0.93Å) and refined as riding, with *U*_iso_(H) = 1.2*U*_eq_(carrier). The water H atoms were located in a difference Fourier map and were refined with distance restraints of O—H = 0.85Å and H···H = 1.35Å. The displacement parameters of the H atoms attached to atom O2W were tied to those of the parent atom by a factor of 1.5, while those on O1W were refined freely.

### Crystal data

**Table:**

[Zn(C_12_H_8_O_4_)(C_12_H_8_N_2_)(H_2_O)]·0.5H_2_O	*Z* = 2
*M**_r_* = 488.78	*F*(000) = 502
Triclinic, *P*1	*D*_x_ = 1.509 Mg m^−^^3^
Hall symbol: -P 1	Mo *K*α radiation, λ = 0.71073 Å
*a* = 8.959 (5) Å	Cell parameters from 3887 reflections
*b* = 11.505 (5) Å	θ = 3.0–25.4°
*c* = 11.691 (5) Å	µ = 1.18 mm^−^^1^
α = 67.219 (5)°	*T* = 293 K
β = 76.434 (5)°	Block, colourless
γ = 89.555 (5)°	0.30 × 0.22 × 0.19 mm
*V* = 1075.4 (9) Å^3^	

### Data collection

**Table:**

Bruker APEX CCD area-detector diffractometer	3887 independent reflections
Radiation source: fine-focus sealed tube	3193 reflections with *I* > 2σ(*I*)
graphite	*R*_int_ = 0.019
φ and ω scans	θ_max_ = 25.4°, θ_min_ = 4.2°
Absorption correction: multi-scan (*SADABS*; Sheldrick 1996)	*h* = −10→9
*T*_min_ = 0.701, *T*_max_ = 0.792	*k* = −11→13
6674 measured reflections	*l* = −12→14

### Refinement

**Table:**

Refinement on *F*^2^	Primary atom site location: structure-invariant direct methods
Least-squares matrix: full	Secondary atom site location: difference Fourier map
*R*[*F*^2^ > 2σ(*F*^2^)] = 0.036	Hydrogen site location: inferred from neighbouring sites
*wR*(*F*^2^) = 0.101	H atoms treated by a mixture of independent and constrained refinement
*S* = 0.98	*w* = 1/[σ^2^(*F*_o_^2^) + (0.0712*P*)^2^] where *P* = (*F*_o_^2^ + 2*F*_c_^2^)/3
3887 reflections	(Δ/σ)_max_ < 0.001
312 parameters	Δρ_max_ = 0.90 e Å^−^^3^
6 restraints	Δρ_min_ = −0.24 e Å^−^^3^

### Special details

**Table:**

Geometry. All esds (except the esd in the dihedral angle between two l.s. planes) are estimated using the full covariance matrix. The cell esds are taken into account individually in the estimation of esds in distances, angles and torsion angles; correlations between esds in cell parameters are only used when they are defined by crystal symmetry. An approximate (isotropic) treatment of cell esds is used for estimating esds involving l.s. planes.
Refinement. Refinement of F^2^ against ALL reflections. The weighted R-factor wR and goodness of fit S are based on F^2^, conventional R-factors R are based on F, with F set to zero for negative F^2^. The threshold expression of F^2^ > 2sigma(F^2^) is used only for calculating R-factors(gt) etc. and is not relevant to the choice of reflections for refinement. R-factors based on F^2^ are statistically about twice as large as those based on F, and R- factors based on ALL data will be even larger.

### Fractional atomic coordinates and isotropic or equivalent isotropic displacement parameters (Å^2^)

**Table:**

	*x*	*y*	*z*	*U*_iso_*/*U*_eq_	Occ. (<1)
Zn1	0.22324 (4)	0.16358 (3)	0.84090 (3)	0.02776 (13)	
O1W	0.1608 (2)	−0.02160 (18)	0.87917 (19)	0.0326 (4)	
O2	0.1060 (3)	0.0767 (2)	1.1643 (2)	0.0512 (6)	
O3	−0.0271 (2)	0.20043 (17)	0.88909 (18)	0.0391 (5)	
O4	0.1579 (2)	0.3439 (2)	0.8484 (2)	0.0469 (5)	
O1	0.3082 (3)	0.1033 (2)	0.99979 (18)	0.0429 (5)	
N2	0.1984 (3)	0.2138 (2)	0.6449 (2)	0.0311 (5)	
N1	0.4515 (3)	0.2360 (2)	0.7226 (2)	0.0310 (5)	
C24	0.0755 (4)	0.1957 (3)	0.6072 (3)	0.0397 (7)	
H24	−0.0132	0.1519	0.6694	0.048*	
C4	0.4100 (3)	0.0176 (2)	1.4115 (3)	0.0312 (6)	
C12	−0.4743 (3)	0.6175 (2)	0.9997 (3)	0.0302 (6)	
H12	−0.4565	0.6976	0.9986	0.036*	
C18	0.3274 (3)	0.2775 (2)	0.5531 (2)	0.0293 (6)	
C16	0.5967 (4)	0.3587 (3)	0.5039 (3)	0.0398 (7)	
C21	0.3323 (4)	0.3264 (3)	0.4225 (3)	0.0385 (7)	
C10	−0.3744 (3)	0.4296 (2)	0.9805 (2)	0.0262 (6)	
C14	0.7138 (4)	0.3119 (3)	0.6779 (4)	0.0529 (9)	
H14	0.7988	0.3171	0.7092	0.064*	
C3	0.3115 (4)	0.0349 (3)	1.3225 (3)	0.0338 (6)	
H3	0.2058	0.0276	1.3575	0.041*	
C2	0.3556 (4)	0.0593 (3)	1.1999 (3)	0.0350 (7)	
H2	0.4604	0.0638	1.1629	0.042*	
C9	−0.2477 (3)	0.3549 (2)	0.9552 (3)	0.0298 (6)	
H9	−0.2753	0.2706	0.9736	0.036*	
C5	0.5685 (4)	0.0473 (3)	1.3708 (3)	0.0398 (7)	
H5	0.6161	0.0793	1.2835	0.048*	
C6	0.6565 (4)	0.0305 (3)	1.4563 (3)	0.0387 (7)	
H6	0.7624	0.0515	1.4259	0.046*	
C11	−0.3509 (3)	0.5488 (2)	0.9820 (2)	0.0303 (6)	
H11	−0.2517	0.5818	0.9709	0.036*	
C17	0.4611 (3)	0.2916 (2)	0.5953 (2)	0.0297 (6)	
C15	0.7236 (4)	0.3696 (3)	0.5503 (4)	0.0506 (9)	
H15	0.8145	0.4161	0.4941	0.061*	
C8	−0.0978 (3)	0.3927 (3)	0.9090 (3)	0.0333 (6)	
H8	−0.0641	0.4748	0.8939	0.040*	
C22	0.2003 (4)	0.3045 (3)	0.3869 (3)	0.0466 (8)	
H22	0.1996	0.3345	0.3009	0.056*	
C23	0.0726 (4)	0.2386 (3)	0.4800 (3)	0.0467 (8)	
H23	−0.0160	0.2227	0.4578	0.056*	
C1	0.2466 (4)	0.0804 (2)	1.1161 (3)	0.0345 (7)	
C7	0.0161 (3)	0.3079 (3)	0.8811 (3)	0.0335 (6)	
C19	0.5968 (4)	0.4111 (3)	0.3712 (3)	0.0491 (9)	
H19	0.6851	0.4579	0.3107	0.059*	
C20	0.4736 (4)	0.3944 (3)	0.3323 (3)	0.0499 (9)	
H20	0.4788	0.4273	0.2450	0.060*	
C13	0.5761 (4)	0.2450 (3)	0.7616 (3)	0.0412 (7)	
H13	0.5717	0.2052	0.8486	0.049*	
O2W	−0.0410 (4)	0.2769 (3)	1.2193 (3)	0.0270 (8)	0.50
HW22	−0.121 (4)	0.275 (5)	1.198 (6)	0.040*	0.50
HW21	0.008 (5)	0.223 (4)	1.202 (6)	0.040*	0.50
HW12	0.073 (4)	−0.019 (4)	0.863 (4)	0.090 (16)*	
HW11	0.144 (4)	−0.074 (3)	0.958 (2)	0.057 (11)*	

### Atomic displacement parameters (Å^2^)

**Table:**

	*U*^11^	*U*^22^	*U*^33^	*U*^12^	*U*^13^	*U*^23^
Zn1	0.0284 (2)	0.03257 (19)	0.02057 (18)	0.00310 (12)	−0.00688 (12)	−0.00830 (13)
O1W	0.0300 (12)	0.0337 (10)	0.0290 (11)	−0.0016 (8)	−0.0075 (9)	−0.0067 (9)
O2	0.0353 (13)	0.0729 (15)	0.0441 (13)	−0.0072 (11)	−0.0161 (10)	−0.0180 (11)
O3	0.0455 (13)	0.0329 (11)	0.0309 (10)	0.0078 (9)	0.0001 (9)	−0.0098 (8)
O4	0.0272 (12)	0.0614 (13)	0.0518 (13)	0.0078 (10)	−0.0054 (10)	−0.0248 (11)
O1	0.0513 (14)	0.0539 (12)	0.0247 (10)	0.0023 (10)	−0.0200 (9)	−0.0108 (9)
N2	0.0305 (13)	0.0360 (12)	0.0228 (11)	−0.0001 (10)	−0.0064 (9)	−0.0075 (10)
N1	0.0310 (13)	0.0360 (12)	0.0299 (12)	0.0063 (10)	−0.0095 (10)	−0.0163 (10)
C24	0.0337 (17)	0.0485 (17)	0.0344 (16)	0.0003 (13)	−0.0104 (13)	−0.0127 (14)
C4	0.0380 (16)	0.0340 (14)	0.0255 (14)	0.0037 (12)	−0.0135 (12)	−0.0127 (11)
C12	0.0329 (15)	0.0255 (13)	0.0314 (14)	0.0005 (11)	−0.0048 (12)	−0.0121 (11)
C18	0.0350 (16)	0.0266 (13)	0.0222 (13)	0.0042 (11)	−0.0046 (11)	−0.0068 (11)
C16	0.0352 (17)	0.0350 (15)	0.0423 (17)	−0.0020 (13)	0.0037 (13)	−0.0156 (13)
C21	0.0473 (19)	0.0384 (15)	0.0242 (14)	0.0083 (13)	−0.0073 (13)	−0.0076 (12)
C10	0.0280 (14)	0.0278 (13)	0.0205 (12)	0.0010 (11)	−0.0044 (11)	−0.0080 (10)
C14	0.0295 (17)	0.077 (2)	0.068 (2)	0.0018 (16)	−0.0132 (16)	−0.044 (2)
C3	0.0356 (16)	0.0383 (15)	0.0292 (15)	0.0011 (12)	−0.0137 (12)	−0.0120 (12)
C2	0.0378 (17)	0.0386 (15)	0.0290 (15)	0.0000 (13)	−0.0164 (13)	−0.0093 (12)
C9	0.0318 (15)	0.0277 (13)	0.0287 (14)	0.0045 (11)	−0.0086 (12)	−0.0091 (11)
C5	0.0402 (18)	0.0584 (18)	0.0192 (13)	0.0004 (14)	−0.0079 (12)	−0.0131 (13)
C6	0.0285 (15)	0.0572 (18)	0.0306 (15)	0.0006 (13)	−0.0088 (12)	−0.0168 (14)
C11	0.0246 (14)	0.0360 (14)	0.0287 (14)	−0.0022 (11)	−0.0028 (11)	−0.0133 (12)
C17	0.0324 (15)	0.0270 (13)	0.0272 (14)	0.0011 (11)	−0.0030 (12)	−0.0105 (11)
C15	0.0322 (18)	0.0564 (19)	0.061 (2)	−0.0060 (15)	0.0019 (15)	−0.0283 (17)
C8	0.0313 (16)	0.0324 (14)	0.0348 (15)	0.0032 (12)	−0.0077 (12)	−0.0121 (12)
C22	0.057 (2)	0.0567 (19)	0.0252 (15)	0.0122 (16)	−0.0152 (15)	−0.0119 (14)
C23	0.046 (2)	0.065 (2)	0.0369 (17)	0.0091 (16)	−0.0225 (15)	−0.0210 (15)
C1	0.0420 (19)	0.0300 (14)	0.0300 (15)	−0.0061 (12)	−0.0169 (13)	−0.0056 (12)
C7	0.0347 (17)	0.0369 (16)	0.0229 (14)	0.0073 (12)	−0.0059 (12)	−0.0065 (12)
C19	0.044 (2)	0.0462 (18)	0.0369 (17)	−0.0047 (15)	0.0089 (15)	−0.0064 (14)
C20	0.059 (2)	0.0505 (18)	0.0210 (14)	0.0018 (16)	0.0035 (15)	−0.0014 (13)
C13	0.0371 (17)	0.0548 (18)	0.0437 (17)	0.0104 (14)	−0.0172 (14)	−0.0282 (15)
O2W	0.029 (2)	0.0325 (19)	0.0288 (19)	0.0005 (15)	−0.0180 (16)	−0.0163 (16)

### Geometric parameters (Å, °)

**Table:**

Zn1—O1	2.041 (2)	C21—C20	1.442 (5)
Zn1—O1W	2.053 (2)	C10—C11	1.396 (4)
Zn1—N1	2.144 (3)	C10—C12^ii^	1.397 (4)
Zn1—O4	2.180 (2)	C10—C9	1.462 (4)
Zn1—N2	2.202 (2)	C14—C15	1.359 (5)
Zn1—O3	2.260 (2)	C14—C13	1.392 (5)
Zn1—C7	2.563 (3)	C14—H14	0.9300
O1W—HW12	0.85 (3)	C3—C2	1.309 (4)
O1W—HW11	0.86 (2)	C3—H3	0.9300
O2—C1	1.246 (4)	C2—C1	1.494 (4)
O3—C7	1.260 (4)	C2—H2	0.9300
O4—C7	1.261 (4)	C9—C8	1.331 (4)
O1—C1	1.263 (4)	C9—H9	0.9300
N2—C24	1.324 (4)	C5—C6	1.369 (4)
N2—C18	1.360 (4)	C5—H5	0.9300
N1—C13	1.320 (4)	C6—C4^i^	1.401 (4)
N1—C17	1.355 (4)	C6—H6	0.9300
C24—C23	1.380 (4)	C11—H11	0.9300
C24—H24	0.9300	C15—H15	0.9300
C4—C5	1.388 (4)	C8—C7	1.475 (4)
C4—C6^i^	1.401 (4)	C8—H8	0.9300
C4—C3	1.472 (4)	C22—C23	1.363 (5)
C12—C11	1.378 (4)	C22—H22	0.9300
C12—C10^ii^	1.397 (4)	C23—H23	0.9300
C12—H12	0.9300	C19—C20	1.329 (5)
C18—C21	1.398 (4)	C19—H19	0.9300
C18—C17	1.431 (4)	C20—H20	0.9300
C16—C15	1.395 (5)	C13—H13	0.9300
C16—C17	1.407 (4)	O2W—HW22	0.815 (10)
C16—C19	1.431 (5)	O2W—HW21	0.816 (10)
C21—C22	1.396 (5)		
			
O1—Zn1—O1W	89.22 (9)	C15—C14—H14	120.1
O1—Zn1—N1	89.76 (9)	C13—C14—H14	120.1
O1W—Zn1—N1	115.47 (8)	C2—C3—C4	127.5 (3)
O1—Zn1—O4	96.13 (9)	C2—C3—H3	116.2
O1W—Zn1—O4	149.07 (9)	C4—C3—H3	116.2
N1—Zn1—O4	95.05 (8)	C3—C2—C1	123.6 (3)
O1—Zn1—N2	162.97 (9)	C3—C2—H2	118.2
O1W—Zn1—N2	88.25 (8)	C1—C2—H2	118.2
N1—Zn1—N2	76.20 (9)	C8—C9—C10	128.0 (3)
O4—Zn1—N2	94.67 (9)	C8—C9—H9	116.0
O1—Zn1—O3	111.13 (9)	C10—C9—H9	116.0
O1W—Zn1—O3	90.77 (8)	C6—C5—C4	121.4 (3)
N1—Zn1—O3	147.13 (8)	C6—C5—H5	119.3
O4—Zn1—O3	58.87 (8)	C4—C5—H5	119.3
N2—Zn1—O3	85.75 (8)	C5—C6—C4^i^	121.3 (3)
O1—Zn1—C7	104.70 (9)	C5—C6—H6	119.3
O1W—Zn1—C7	119.90 (9)	C4^i^—C6—H6	119.3
N1—Zn1—C7	122.57 (9)	C12—C11—C10	120.2 (3)
O4—Zn1—C7	29.45 (9)	C12—C11—H11	119.9
N2—Zn1—C7	91.12 (9)	C10—C11—H11	119.9
O3—Zn1—C7	29.44 (8)	N1—C17—C16	123.0 (3)
Zn1—O1W—HW12	104 (3)	N1—C17—C18	117.7 (2)
Zn1—O1W—HW11	116 (2)	C16—C17—C18	119.2 (3)
HW12—O1W—HW11	104 (3)	C14—C15—C16	119.7 (3)
C7—O3—Zn1	88.74 (18)	C14—C15—H15	120.1
C7—O4—Zn1	92.34 (18)	C16—C15—H15	120.1
C1—O1—Zn1	132.4 (2)	C9—C8—C7	121.7 (3)
C24—N2—C18	117.9 (2)	C9—C8—H8	119.1
C24—N2—Zn1	128.96 (19)	C7—C8—H8	119.1
C18—N2—Zn1	113.08 (18)	C23—C22—C21	119.1 (3)
C13—N1—C17	118.0 (3)	C23—C22—H22	120.4
C13—N1—Zn1	126.8 (2)	C21—C22—H22	120.4
C17—N1—Zn1	114.67 (18)	C22—C23—C24	119.6 (3)
N2—C24—C23	123.2 (3)	C22—C23—H23	120.2
N2—C24—H24	118.4	C24—C23—H23	120.2
C23—C24—H24	118.4	O2—C1—O1	125.7 (3)
C5—C4—C6^i^	117.2 (3)	O2—C1—C2	118.7 (3)
C5—C4—C3	123.0 (3)	O1—C1—C2	115.5 (3)
C6^i^—C4—C3	119.8 (3)	O3—C7—O4	120.0 (3)
C11—C12—C10^ii^	121.8 (2)	O3—C7—C8	120.6 (3)
C11—C12—H12	119.1	O4—C7—C8	119.4 (3)
C10^ii^—C12—H12	119.1	O3—C7—Zn1	61.82 (15)
N2—C18—C21	122.3 (3)	O4—C7—Zn1	58.21 (16)
N2—C18—C17	117.1 (2)	C8—C7—Zn1	176.5 (2)
C21—C18—C17	120.6 (3)	C20—C19—C16	121.5 (3)
C15—C16—C17	116.9 (3)	C20—C19—H19	119.2
C15—C16—C19	124.2 (3)	C16—C19—H19	119.2
C17—C16—C19	118.9 (3)	C19—C20—C21	121.4 (3)
C22—C21—C18	117.9 (3)	C19—C20—H20	119.3
C22—C21—C20	123.8 (3)	C21—C20—H20	119.3
C18—C21—C20	118.3 (3)	N1—C13—C14	122.6 (3)
C11—C10—C12^ii^	118.0 (2)	N1—C13—H13	118.7
C11—C10—C9	122.8 (2)	C14—C13—H13	118.7
C12^ii^—C10—C9	119.1 (2)	HW22—O2W—HW21	105.6 (18)
C15—C14—C13	119.8 (3)		

Symmetry codes: (i) −*x*+1, −*y*, −*z*+3; (ii) −*x*−1, −*y*+1, −*z*+2.

### Hydrogen-bond geometry (Å, °)

**Table:**

*D*—H···*A*	*D*—H	H···*A*	*D*···*A*	*D*—H···*A*
O1W—HW12···O2^iii^	0.85 (3)	1.87 (3)	2.687 (3)	158 (4)
O1W—HW11···O3^iii^	0.86 (2)	1.88 (3)	2.685 (3)	155 (3)
O2W—HW21···O2	0.82 (1)	2.04 (1)	2.856 (5)	173 (5)

Symmetry codes: (iii) −*x*, −*y*, −*z*+2.
